# Taxonomic revision of the Afrotropical *Agabus raffrayi* species group with the description of four new species (Coleoptera, Dytiscidae)

**DOI:** 10.3897/zookeys.963.53470

**Published:** 2020-08-24

**Authors:** William F. Englund, Laban Njoroge, Olof Biström, Kelly B. Miller, David T. Bilton, Johannes Bergsten

**Affiliations:** 1 Swedish Museum of Natural History, Department of Zoology, Box 50007, SE-10405 Stockholm, Sweden Swedish Museum of Natural History Stockholm Sweden; 2 National Museums of Kenya, Section of Invertebrate Zoology, Museum Hill, P.O. BOX 40658- 00100, Nairobi, Kenya National Museums of Kenya Nairobi Kenya; 3 Finnish Museum of Natural History, Zoology Unit, P.O. Box 17, FI-00014 University of Helsinki, Finland Finnish Museum of Natural History Helsinki Finland; 4 Department of Biology and Museum of Southwestern Biology, University of New Mexico, Albuquerque, NM 87131-0001, USA University of New Mexico Albuquerque United States of America; 5 Marine Biology and Ecology Research Centre, School of Biological and Marine Sciences, University of Plymouth, Drake Circus, Plymouth PL4 8AA, UK University of Plymouth Plymouth United Kingdom; 6 Department of Zoology, University of Johannesburg, PO Box 524, Auckland Park, Johannesburg 2006, South Africa University of Johannesburg Johannesburg South Africa

**Keywords:** Afromontane, diving beetles, freshwater, new species, taxonomy

## Abstract

We revise the Afrotropical *Agabus raffrayi* species group, motivated by the discovery of new diversity in Kenya and South Africa. Whilst *Agabus* is mainly a holarctic genus, the *Agabus raffrayi* group is restricted to high altitude regions of eastern Africa and temperate parts of South Africa, from where we describe the southernmost *Agabus* in the world. The following new species are introduced: *Agabus anguluverpus***sp. nov.** from Mount Kenya in central Kenya, *Agabus austellus***sp. nov.** a widespread species in South Africa, *Agabus riberae***sp. nov.** from the Kamiesberg and northeastern Cederberg ranges in the Northern and Western Cape Provinces of South Africa and *Agabus agulhas***sp. nov.** from the Agulhas Plain, Western Cape Province, South Africa. We provide a distribution map, a determination key for males, quantitative measurements of diagnostic characters, habitus photos and detailed photos of male genitalia for all described species in the group, as well as images of diagnostic characters and habitats. The presence or absence of an elongated section between the subapical broadening and the base of the apical and subapical teeth of the male aedeagus is a useful novel character, first revealed by our study. In contrast with the most recent revision of Afrotropical *Agabus*, we show that *Agabus ruwenzoricus* Guignot, 1936 is restricted to eastern Africa; South African records of this species having been based on misidentifications, no species of the group being common to southern and eastern Africa. We speculate that the *raffrayi* group may display phylogenetic niche conservatism, being restricted, as an originally temperate taxon, to higher elevations in tropical eastern Africa, but occurring at lower altitudes in temperate South Africa.

## Introduction

With over 170 species, *Agabus* Leach, 1817 is a large and complex genus of diving beetles which is particularly diverse in temperate regions of the Holarctic. Species-level identification characters in *Agabus* are often subtle, with the taxa known from the Afrotropical region being no exception. Nilsson (1992a) made a significant contribution to our understanding of Afrotropical *Agabus*, defining species groups and providing keys for the identification of all species known at the time. Afrotropical *Agabus* are mainly high altitude specialists, distributed from Ethiopia to South Africa. There are currently 17 described species, placed in four distinct species groups, the *ambulator*, *ragazzi*, *cordatus* and *raffrayi* groups (Nilsson 1992a). With the exception of some species in the *raffrayi* group, all Afrotropical taxa are endemic to Ethiopia (Nilsson 1992a). Recently, a combination of newly collected material together with problems encountered with certain morphological characters have prompted us to conduct a revision of the *Agabus raffrayi* group. At present this group consists of five species: *A. dytiscoides* Régimbart, 1908, *A. pallidus* Omer-Cooper, 1931, *A. raffrayi* Sharp, 1882, *A. ruwenzoricus* Guignot, 1936 and *A. sjostedti* Régimbart, 1908. This revision aims to clarify morphological species delimitations for these taxa, describe four new species which have come to light more recently, and present a new identification key for the group.

**Figure 1.**
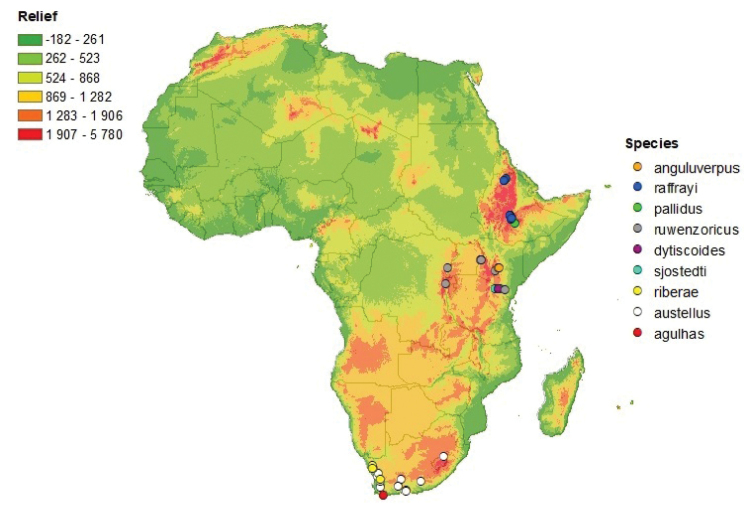
Distribution map of the *Agabus raffrayi* group. Relief is expressed in meters above sea level.

With the exception of the three taxa described here from South Africa, species of the *Agabus raffrayi* group are apparently restricted to relatively high elevations, between 1900 and 4300 m (see Fig. 1). The high number of montane specialists in the group could be related to the environmental history of the region. African tropico-alpine ecosystems are believed to be relatively young (Linder 2014) and Nilsson (1992a) suggested that the cold, dry climate of the Quaternary may have forced Afrotropical *Agabus* to adapt to higher altitudes in order to survive, and that this adaptation cannot easily be reversed. Additionally, species of this largely temperate genus may display a degree of phylogenetic niche conservatism (sensu Morinière et al. 2016), physiologically restricting them to relatively cool climates. Such suggestions correlate with the apparent lack of high-altitude specialist *Agabus* in South Africa, an area that has retained a relatively temperate climate throughout the Quaternary period, particularly in the Cape (Meadows and Baxter 1999; Dupont et al. 2011).

## Material and methods

### Abbreviations

Material studied is deposited in the following collections:

**AMG** Albany Museum, Grahamstown, South Africa

**BMNH** The Natural History Museum, London, UK

**CBP** Collection D T Bilton, Plymouth, UK

**IBE** Institut de Biologia Evolutiva, Barcelona, Spain

**ISAM** Iziko South African Museum, Cape Town, South Africa

**MfN** Museum für Naturkunde der Humboldt-Universität, Berlin, Germany

**MZLU** Biological Museum, Entomological collections, Lund, Sweden

**NHRS** Swedish Museum of Natural History, Stockholm, Sweden

**NMK** National Museums of Kenya, Nairobi, Kenya

**SANC** South African National Collection of Insects, Pretoria, South Africa

**ZSM** Zoologische Staatssammlung, München, Germany

Additional acronyms used:

**WC** Width of metacoxal plate

**WS** Width of metasternal wing (correct term is lateral extension of the metaventrite but we use here the term “metasternal wing” to adhere to previous literature on the group, e.g., Nilsson and Persson 1990).

### Measurements

Characters were measured using a WILD 445111 10x/21B ocular on a LEICA M125 microscope.

Individual measurements were taken as follows:

Metatarsomeres 2 and 5 were measured from a lateral perspective, using the maximum length and width.

**Figure 2.**
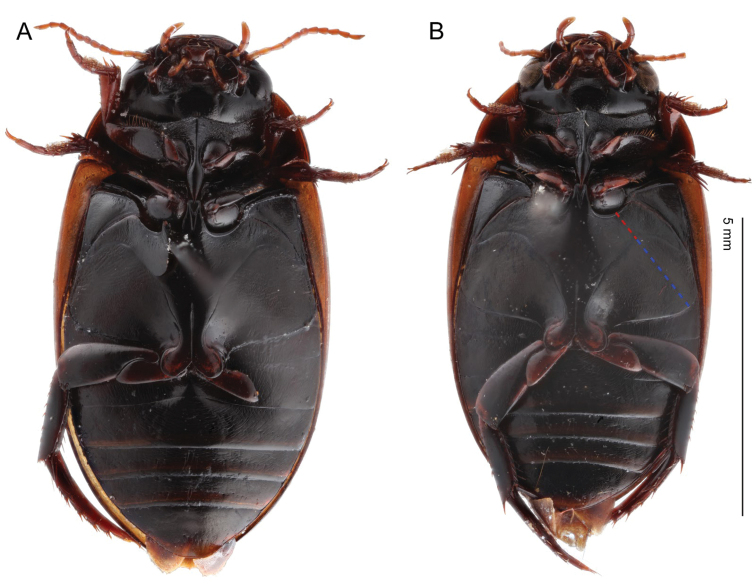
*Agabus* spp., metasternal wing and metacoxal plate in ventral view **A***A. pallidus***B***A. raffrayi*. Dashed lines indicate measurements used; red: metasternal wing (WS), blue: metacoxal plate (WC).

The ratio of the width of metacoxal plate to the width of metasternal wing (WC/WS) was measured as in Nilsson and Persson (1990): WS was measured at the shortest distance between the mesocoxa and the metacoxal plate, with WC continuing along the line of WS (see Fig. 2).

**Figure 3.**
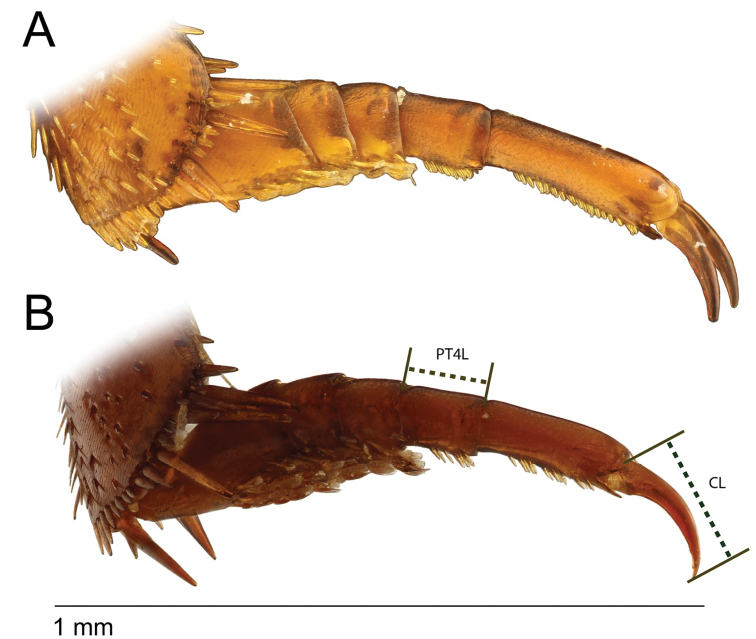
*Agabus* spp., protarsomeres in anterior view **A***A. dytiscoides***B***A. raffrayi*. PT4L = measured distance for length of protarsomere 4; CL = measured distance for length of protarsal claw.

Protarsal claw/protarsomere 4. The length of the protarsal claw was measured from a lateral perspective, using the maximum distance between the base and apex of the claw (see Fig. 3); maximum length of protarsomere 4 was also measured from a lateral perspective.

**Figure 4.**
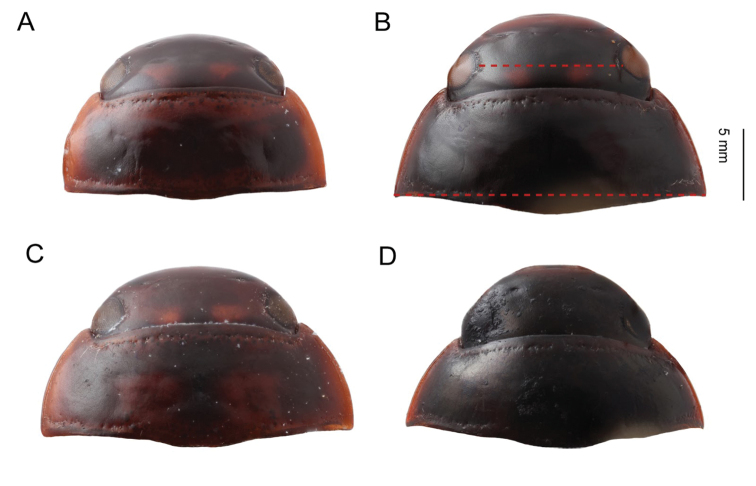
*Agabus* spp., head and pronotum in dorsal view **A***A. dytiscoides***B***A. ruwenzoricus***C***A. sjostedti***D***A. pallidus*. Red dashed lines indicate measurements used for interocular distance and pronotal width.

Pronotum/Interocular distance was measured in dorsal view, using the maximum distance for pronotum width and minimum interocular distance (see Fig. 4).

Body length was measured in dorsal view, from the anterior margin of the head to the tip of the elytra.

### Preparation of genitalia

Male genitalia were extracted from the tip of the abdomen using fine forceps. The aedeagus and parameres were then carefully separated from the last divided sternite (VIII) and glued onto a mounting card on the same pin as the specimen. The removed sternites of the abdomen as well as parts removed during extraction of genitalia were also mounted on the same card. Dry specimens were submerged in hot water for 15 minutes prior to preparation in order to soften the body to facilitate extraction.

### Photographs, figures and tables

Photographs were taken using a Canon EOS 5D Mark II DSLR camera with a Canon MP-E 65 mm 1–5× macro lens mounted on a motorized rail (Cognisys Stackshot). Elytral microreticulation was imaged using a Canon EOS 600D camera attached to a Leica Z6 APO macroscope with a 2× objective lens. Aedeagal apices of South African species were imaged with the same system, as temporary mounts in hand sanitizer gel. Images were stacked using the PMax algorithm in Zerene Stacker and manually edited in Photoshop. Boxplots were made using R version 3.4.3.

## Taxonomic results

### *Agabus raffrayi* group

Nilsson (1992a) gives a full diagnosis of the *Agabus raffrayi* group. It is noteworthy that Nilsson was unable to find a single synapomorphic character for the group, but states that the very similar appearance of the aedeagus amongst the species of the group might suggest a common evolutionary history. The aedeagus shape of the four new species described here does differ somewhat from the five species known to Nilsson, but follows the same basic design. Nilsson (1992b) described larval morphology of some species belonging to the *raffrayi*, *ambulator* and *ragazzi* groups. He concluded that *A. raffrayi* (and probably also *A. ruwenzoricus*) larvae can be distinguished from the two other groups by its short urogomphus as well as terga with long spiniform setae (among other characters).

The nine species of the group recognised in this revision are all endemic to the Afrotropical region (see Fig. 1). Three species are endemic to the Republic of South Africa, one of which is widespread there. Two species are endemic to Ethiopia, two species endemic to Tanzania, and one endemic to Kenya. Based on current understanding, only one of the nine species has a geographical range spanning over several countries, namely *A. ruwenzoricus*, collected in Kenya, Rwanda, Uganda and the Democratic Republic of the Congo.

### Key to males of the *Agabus raffrayi* group

**Table:** 

1	Aedeagus not prolonged between subapical broadening and base of apical and subapical teeth (as in Figs 5D, 6)	**2**
–	Aedeagus prolonged between subapical broadening and base of apical and subapical teeth (as in Fig. 5A–C)	**5**
2	Pronotal bead broad, especially anteriorly. Aedeagus in ventral view with apex straight (Fig. 7A), in lateral view evenly thickened and not distinctly broadened subapically; subapical tooth slightly angled both at base and apex (see Fig. 8F). Females with coarse microreticulation on pronotum and elytra, much coarser than in males. Known only from Mt. Kenya	***A. anguluverpus* sp. nov.**
–	Pronotal bead narrower and not becoming broader anteriorly. Aedeagus in ventral view with apex asymmetrically curved (see Fig. 7B), in lateral view usually subapically broadened (see Fig. 8G–I); subapical tooth not distinctly angled twice. Female microreticulation much more similar to males. South Africa	**3**
3	Base of subapical tooth of aedeagus distinctly angled (Figs 6G, 8I). Scutellum lighter than elytra	***A. agulhas* sp. nov.**
–	Base of subapical tooth of aedeagus not distinctly angled (Figs 6A–F, 8G–H). Scutellum same colour as elytra	**4**
4	Metasternal wing narrow, WC/WS > 3.1 in all specimens. Microreticulation of elytral disc dominated by relatively small, approximately isodiametric meshes in most specimens (Fig. 9B, C)	***A. austellus* sp. nov.**
–	Metasternal wing wide, WC/WS < 3.0 in most specimens. Microreticulation of elytral disc dominated by relatively large, uneven meshes (Fig. 9E)	***A. riberae* sp. nov.**
5	Hypomeron broadly visible in strict lateral view (see Fig. 10A, B). Protarsal claw < 1.6× as long as protarsomere 4. Known only from high mountains of Tanzania	**6**
–	Hypomeron not visible in strict lateral view (see Fig. 10C, D). Protarsal claw usually > 1.6× as long as protarsomere 4. Known from Kenya, Rwanda, the Democratic Republic of the Congo, Uganda and Ethiopia	**7**
6	Large species, body length > 8 mm. Pronotum broad, width of pronotum > 2× interocular distance. Metasternal wing broad, WC/WS > 3.6 in most specimens. Subapical tooth of aedeagus robust, with tip angled downwards	***A. sjostedti***
–	Smaller species, body length < 8 mm long. Pronotum narrow, width of pronotum < 2× interocular distance. Metasternal wing narrow, WC/WS < 3.6 in most specimens. Subapical tooth of aedeagus less robust, with tip not distinctly angled downwards	***A. dytiscoides***
7	Interocular spots clearly visible (as in Fig. 4B). Known from Kenya, Rwanda, the Democratic Republic of the Congo and Uganda	***A. ruwenzoricus***
–	Specimens normally without interocular spots (as in Fig. 4D). Ethiopia	**8**
8	Metasternal wing narrow, WC/WS > 3.0 in most specimens	***A. pallidus***
–	Metasternal wing broad, WC/WS < 2.9 in most specimens	***A. raffrayi***

**Figure 5.**
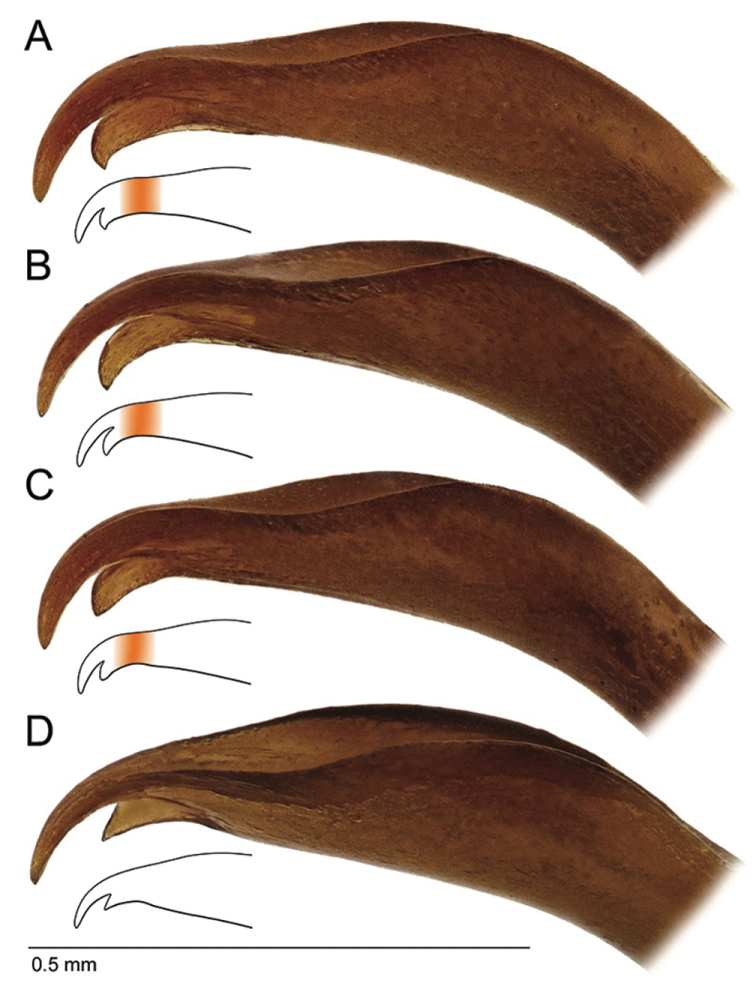
*Agabus* spp., lateral view of tip of aedeagus **A–C***A. raffrayi*, different specimens showing the variation in shape of the subapical tooth **D***A. austellus* sp. nov. Note the prolonged section between subapical broadening and base of apical and subapical teeth in *A. raffrayi* (indicated with orange in the smaller outline illustrations).

**Figure 6.**
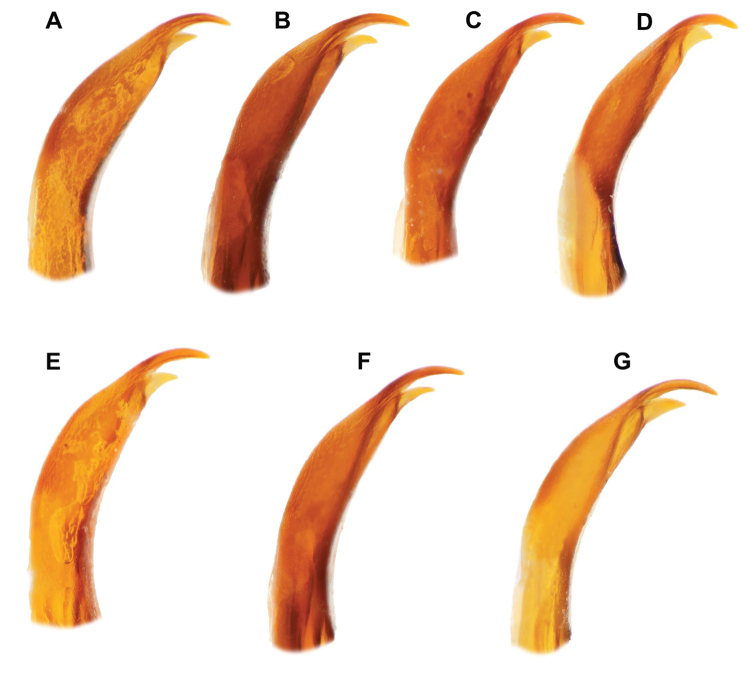
South African *Agabus* spp., aedeagal apices, lateral view **A***A. austellus* sp. nov. holotype, Keurboom, Western Cape **B***A. austellus* sp. nov., Gydo Pass, Western Cape **C***A. austellus* sp. nov. Groote Swartberg, Western Cape **D***A. austellus* sp. nov. Sentinel Peak, KZN Drakensberg **E***A. riberae* sp. nov. holotype, Kamiesberg, Northern Cape; **F***A. riberae* sp. nov. paratype, Kamiesberg, Northern Cape **G***A. agulhas* sp. nov. holotype, Rattelrivier, Western Cape.

**Figure 7.**
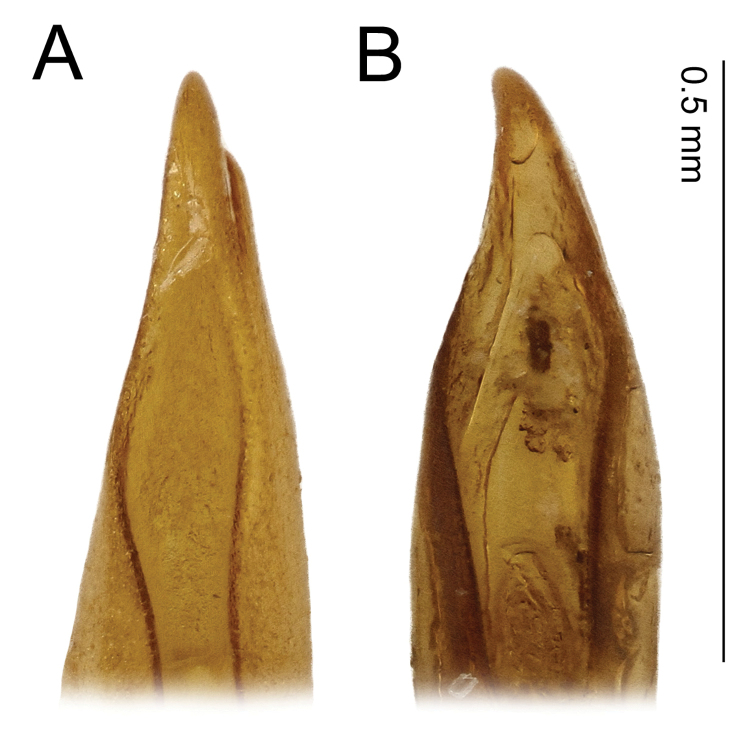
*Agabus* spp., ventral view (following Miller and Nilsson 2003) of apex of aedeagus **A***A. anguluverpus* sp. nov. **B***A. austellus* sp. nov.

#### Agabus raffrayi

Taxon classificationAnimaliaColeopteraDytiscidae

Sharp, 1882

2750ED0D-FA48-5C54-8707-87F934361C04

Figures 1 , 2B , 3B , 5A–C , 8D , 10D , 11D , 11I , 12 , 13 , 14

Agabus raffrayi Sharp, 1882: 501–502
Agabus limbicollis Régimbart, 1905: 224–225 (Syn. Nilsson 1992a)

##### Type locality.

*raffrayi* “Abyssinia” [Ethiopia]; *limbicollis* “Abyssinie: Auato, au bord du Nil Bleu, dans le Gindeberat” [Ethiopia: Auato, on the banks of the Blue Nile, in the Gindeberat].

##### Type material.

***Lectotype*** ♂ of *raffrayi* (BMNH) labelled: “♂ Abyssinia, Raffray 782”, “Type”, “Sharp Coll 1905-313.”, “Type 782 Agabus Raffrayi n.sp. Abyssinia”, “LECTOTYPUS ♂ Agabus raffrayi Sharp, 1882 Des. A.Nilsson, 1989”. ***Lectotype*** ♂ of *limbicollis* (MNB) labelled: “N.O. – Africa, Schoa, Falle O. Neumann S.”, “610 Falle”, “Agabus limbicollis Rég. Type.”, “LECTOTYPUS ♂ Agabus limbicollis Régimbart, 1905. Des. Nilsson -90”.

##### Diagnosis.

With a prolonged preapical section of male aedeagus and a pronotal hypomeron which is not visible in lateral view, this species is most similar to *A. pallidus* and *A. ruwenzoricus*. From the former it is separated by its broader metasternal wing (Figs 2, 12) and from the latter by the lack of interocular spots (compare Fig. 4D, B).

##### Description.

Habitus as in Fig. 11D, I.

***Colour***: Head black, most specimens with a small rufous anterior area, interocular spots not present. Pronotum black with rufous margins. Elytra rufotestaceous to brown. Ventral surface black, hypomeron and epipleuron testaceous. Legs rufous to rufopiceous. Antennae and palpi testaceous to rufotestaceous.

***Microreticulation***: Medium impressed on head, pronotum and elytra, similar in both sexes. Composed of a mixture of small and somewhat larger, uneven meshes.

***Structural features***: Body length: 6.96–8.24 mm (see Table 1). Hypomeron not visible in strict lateral view (Fig. 10D), lateral bead of pronotum well defined (see Fig. 10D). Metasternal wing broad, WC/WS less than 2.9 (see Table 1, Figs 2B, 12). Pronotum broad, more than twice as broad as interocular distance (see Table 1, as in Fig. 4B).

***Legs***: Protarsal claws long, > 1.6× as long as protarsomere 4 in all males and most females (see Table 2, Fig. 14). Metatarsomeres short and broad; metatarsomere 2 < 1.6× as long as broad (see Table 2), metatarsomere 5 < 3.0 times as long as broad (see Table 2).

**Figure 8.**
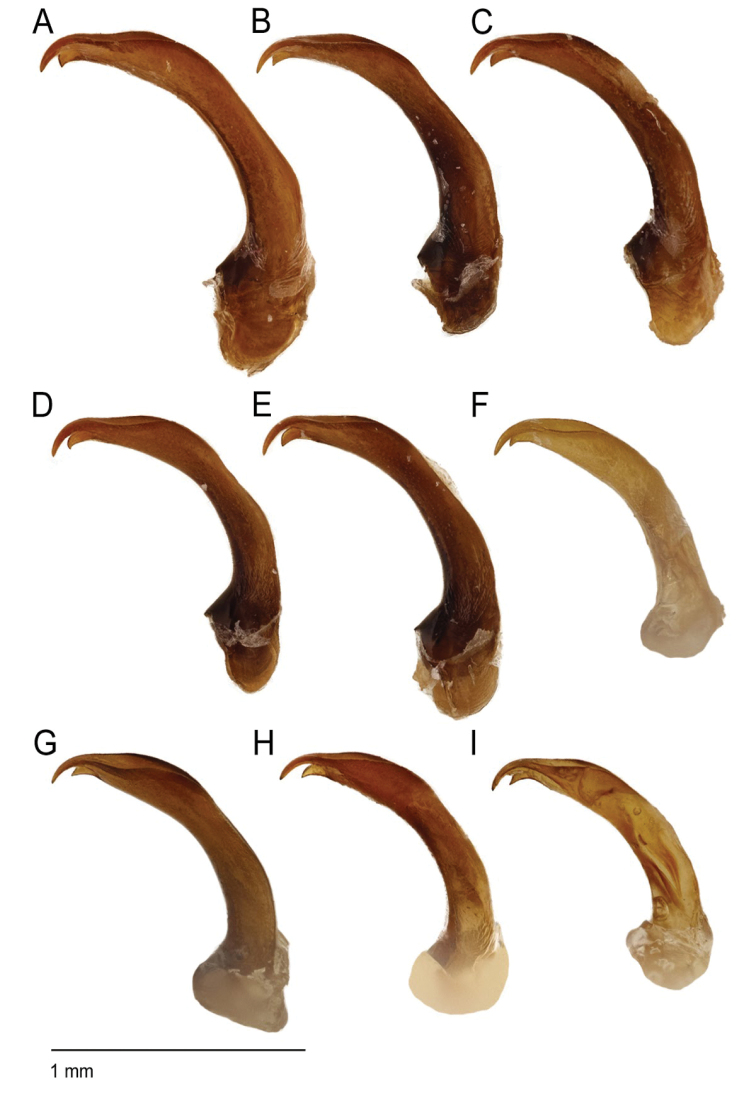
*Agabus* spp., aedeagus in lateral view **A***A. sjostedti***B***A. dytiscoides***C***A. ruwenzoricus***D***A. raffrayi***E***A. pallidus***F***A. anguluverpus* sp. nov. **G***A. austellus* sp. nov. **H***A. riberae* sp. nov. **I***A. agulhas* sp. nov.

***Male genitalia***: Subapically broadened, and prolonged between the subapical broadening and the apical and subapical teeth. Subapical tooth with quite variable shape (see Figs 8D, 5A–C).

**Female**: Externally similar to males but colour of the elytra tends to be slightly lighter.

##### Distribution.

Ethiopia (see Fig. 1). Rocchi (1975) listed the distribution of *A. raffrayi* to also include the Democratic Republic of the Congo, Uganda, Rwanda, Tanzania, Zimbabwe, and South Africa but these specimens are likely to belong to other species.

##### Habitat.

Found in small, often temporary, streams and pools in streambeds at elevations between 2100 to 3200 m (Nilsson and Persson 1990, 1993; Nilsson 1992a).

##### Etymology.

The name refers to the collector of the type specimens, Achille Raffray. The name of the synonym *A. limbicollis* refers to the well-defined lateral bead of the pronotum (Latin: *limbus* = border, *collum* = neck).

##### Comments.

The fact that *A. raffrayi* and *A. pallidus* are distinguishable only on the width of the metasternal wing led some previous authors to suggest the occurrence of a single species which was dimorphic with regard to this character (Jackson 1956). Nilsson and Persson (1990) provided a detailed account of this argument, analysed a large series of specimens and concluded that the variation should rather be interpreted as two separate species. We agree with this assessment and concur that male genitalia are not diagnostic for these two species, only the width of the metasternal wing being reliable. In our measurements, the pronotum is marginally broader in *A. pallidus* but the small sample size forbids any strong conclusions at present (Fig. 13).

Nilsson (1992b) described the larval morphology of *Agabus raffrayi* along with some representatives of two other Afrotropical *Agabus* groups.

**Figure 9.**
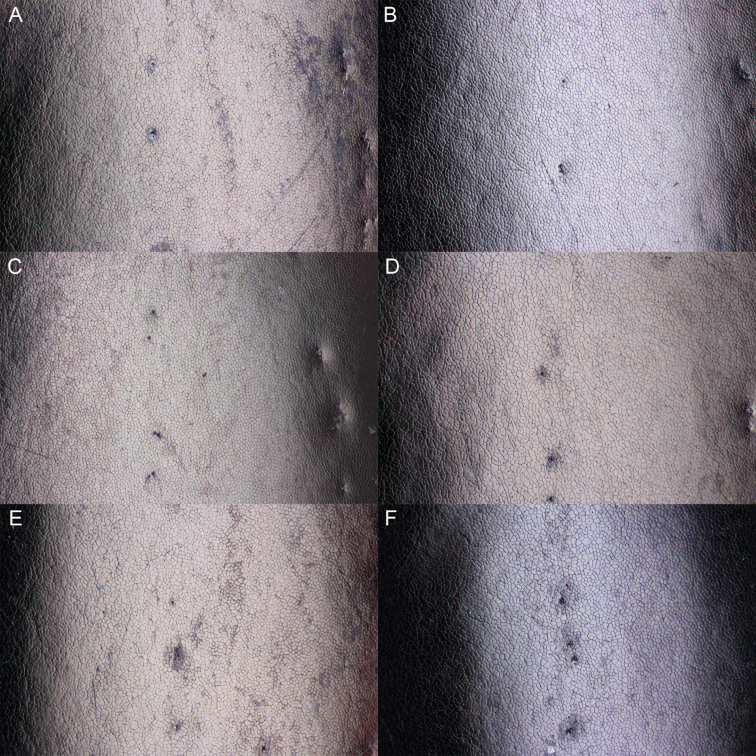
South African *Agabus* spp., microreticulation of male elytral disc **A***A. austellus* sp. nov. holotype, Keurboom, Western Cape **B***A. austellus* sp. nov. Groote Swartberg, Western Cape **C***A. austellus* sp. nov. Sentinel Peak, KZN Drakensberg **D***A. austellus* sp. nov. Harkerville Forest, Western Cape **E***A. riberae* sp. nov. Kamiesberg, Northern Cape **F***A. agulhas* sp. nov. holotype, Rattelrivier, Western Cape.

**Figure 10.**
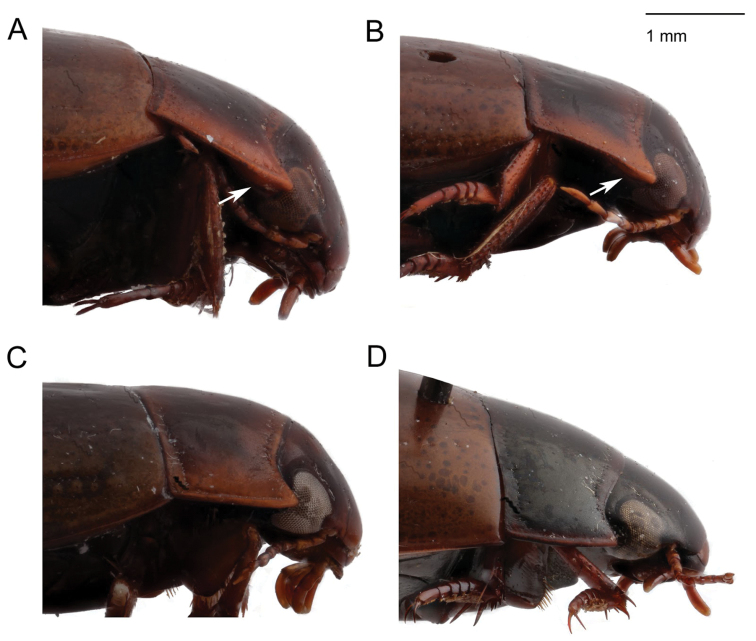
*Agabus* spp., hypomeron in lateral view **A***A. sjostedti***B***A. dytiscoides***C***A. anguluverpus* sp. nov. **D***A. raffrayi*. White arrows indicate hypomeron.

**Table 1.** Morphological characters in the *Agabus raffrayi* group. WC/WS = width of metacoxal plate /width of metasternal wing, PW/ID = width of pronotum/interocular distance, TL = total body length, N = number of specimens, Min = minimum value, Max = maximum value, SD = standard deviation, NA = not applicable.

Species	Sex	WC/WS					PW/ID					TL				
		N	Min	Max	Mean	SD	N	Min	Max	Mean	SD	N	Min	Max	Mean	SD
*A. sjostedti*	♀	2	3.60	3.69	3.64	0.06	2	2.05	2.10	2.07	0.04	2	8.08	8.24	8.16	0.11
	♂	4	3.29	3.65	3.55	0.17	4	2.07	2.24	2.13	0.08	4	8.40	9.12	8.70	0.31
*A. dytiscoides*	♀	2	3.00	3.27	3.13	0.19	2	1.93	1.93	1.93	0.00	2	7.36	7.60	7.48	0.17
	♂	5	2.88	3.40	3.14	0.22	5	1.93	1.98	1.96	0.02	5	7.36	7.76	7.55	0.18
*A. anguluverpus* sp. nov.	♀	1	3.47	3.47	3.47	NA	1	2.10	2.10	2.10	NA	1	7.52	7.52	7.52	NA
	♂	2	3.19	3.53	3.36	0.25	2	2.05	2.15	2.10	0.07	2	7.36	7.44	7.40	0.06
*A. austellus* sp. nov.	♀	17	3.11	4.15	3.55	0.23	17	2.12	2.30	2.22	0.05	17	6.80	8.16	7.53	0.40
	♂	27	3.17	4.0	3.52	0.25	27	2.12	2.49	2.28	0.08	27	7.04	8.40	7.67	0.36
*A. ruwenzoricus*	♀	9	2.90	3.41	3.16	0.19	9	2.05	2.33	2.19	0.10	9	7.36	8.08	7.67	0.21
	♂	17	2.73	3.63	3.05	0.28	17	2.09	2.33	2.24	0.07	17	7.52	8.08	7.86	0.20
*A. raffrayi*	♀	5	2.11	2.84	2.58	0.28	5	2.16	2.33	2.25	0.06	5	6.96	8.16	7.70	0.45
	♂	7	2.46	2.71	2.61	0.10	7	2.25	2.29	2.27	0.01	7	7.52	8.24	7.82	0.31
*A. pallidus*	♀	6	3.06	3.53	3.29	0.16	6	2.15	2.29	2.22	0.05	6	7.52	8.08	7.79	0.25
	♂	5	3.05	3.81	3.39	0.27	5	2.30	2.45	2.34	0.06	5	7.92	8.40	8.14	0.18
*A. riberae* sp. nov.	♀	6	3.00	3.17	3.05	0.07	6	2.24	2.33	2.27	0.03	6	7.21	8.08	7.70	0.35
	♂	16	2.65	3.53	2.91	0.19	16	2.21	2.37	2.28	0.05	16	7.21	8.24	7.83	0.29
*A. agulhas* sp. nov.	♀	2	3.67	3.75	3.71	0.06	2	2.15	2.18	2.16	0.02	2	7.94	7.94	7.94	0
	♂	4	3.67	4.54	4.01	0.38	4	2.10	2.24	2.17	0.06	4	7.60	8.00	7.86	0.19

#### Agabus pallidus

Taxon classificationAnimaliaColeopteraDytiscidae

Omer-Cooper, 1931

43307977-49A1-5093-92E4-2EDDEA934577

Figures 1 , 2A , 3B , 4D , 8E , 11E , 11J , 12 , 13 , 14

Agabus pallidus Omer-Cooper, 1931: 786–787, fig. 3a, pl. 9: 7

##### Type locality.

“Between Addis Abeba and Addis Alem” [Ethiopia].

##### Type material.

***Lectotype*** ♀ (BMNH) labelled: “Type”, “Abyssinia: Between Addis Abeba and Addis Alem 7,500 ft. 18.ix.1926. J.Omer-Cooper.”, “Agabus pallidus, Joyce Omer-Cooper. 1931. TYPE.”, “A. pallidus.O.C.”, “LECTOTYPUS ♀ Agabus pallidus Omer-Cooper, 1931 Des. A. Nilsson, 1989”.

##### Diagnosis.

Similar in all diagnostic features to *A. raffrayi* except for the narrower metasternal wing (Table 1, Figs 2, 12).

##### Description.

**Figure 11.**
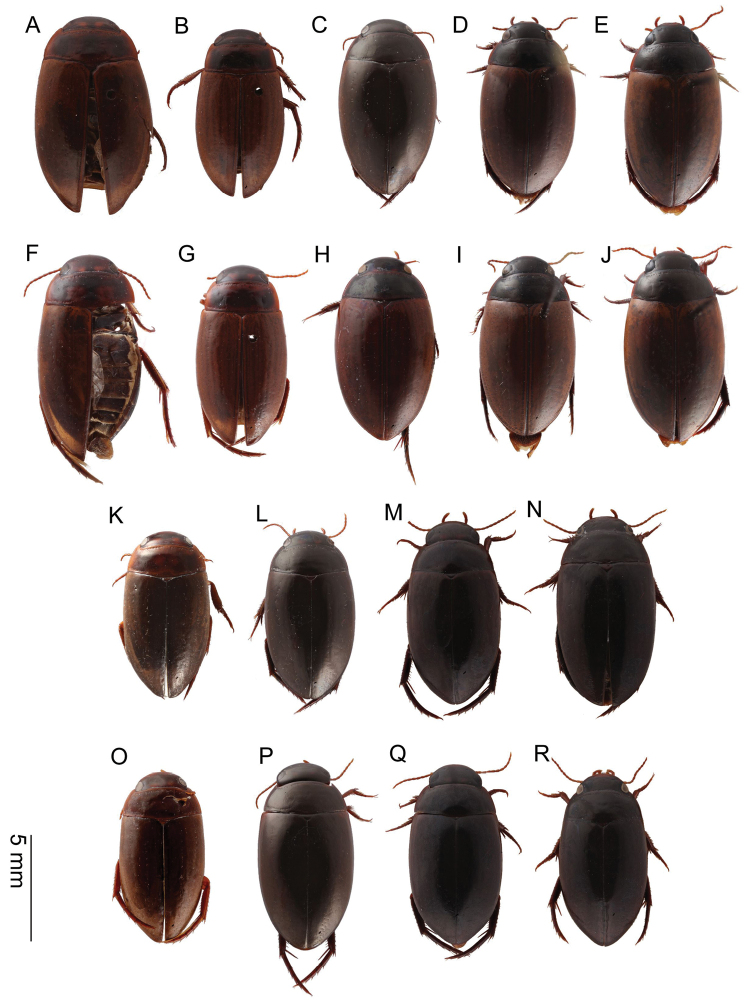
*Agabus* spp., habitus of males in dorsal view **A, F***A. sjostedti***B, G***A. dytiscoides*; **C, H***A. ruwenzoricus***D, I***A. raffrayi***E, J***A. pallidus***K, O***A. anguluverpus* sp. nov. **L, P***A. austellus* sp. nov. **M, Q***A. riberae* sp. nov. **N, R***A. agulhas* sp. nov.

Habitus as in Fig. 11E, J.

***Colour***: Head black, most specimens with a small rufous anterior area, interocular spots not present. Pronotum black with rufous margins. Elytra rufotestaceous to brown. Ventral surface black, hypomeron rufous, epipleuron testaceous to rufotestaceous. Legs rufous to rufopiceous. Antennae and palpi testaceous to rufotestaceous.

***Microreticulation***: Medium impressed on head and pronotum, similar in both sexes. Composed of a mixture of small and somewhat larger, uneven meshes. Elytral microreticulation similar, but less strongly impressed and more uneven, with some relatively elongate meshes, especially close to suture. One female examined (Ethiopia, Arsi, 13 km E Bekoji) has more strongly impressed elytral reticulation, with distinctly wider grooves between meshes.

**Figure 12.**
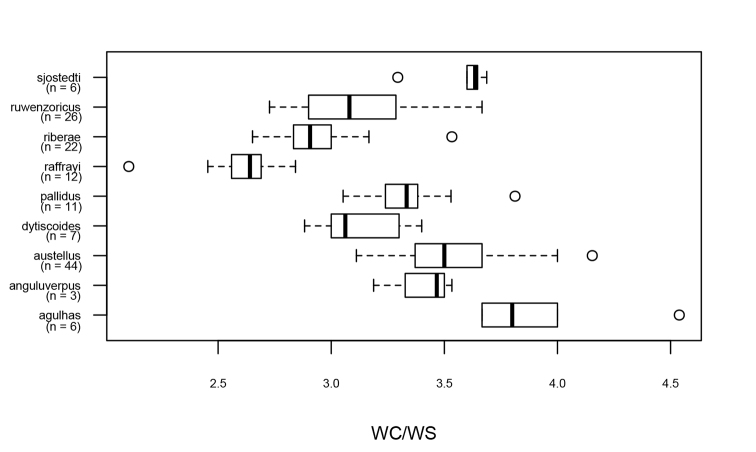
The ratio between width of the metacoxal plate and metasternal wing (WC/WS) in the *A. raffrayi* group (incl. specimens of both sexes). Thick black line inside boxes represents medians, left and right box borders 25^th^ (Q1) and 75^th^ (Q3) percentiles respectively. Whiskers were calculated with the boxplot.stats function in R using the default coefficient value of 1.5 (drawn to the highest and lowest value within 1.5*IQD (Inter Quartile Distance = Q3-Q1) away from the 75^th^ and 25^th^ percentiles respectively). Note that this character fully separates *A. raffrayi* from *A. pallidus*.

**Figure 13.**
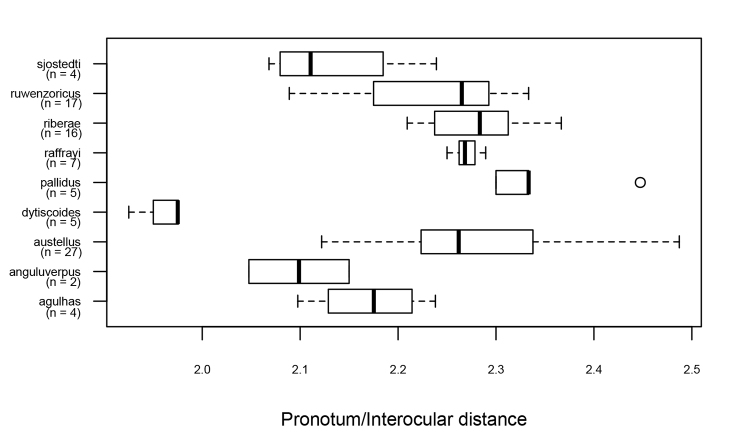
The ratio between pronotal width and interocular distance in males of the *Agabus raffrayi* group. Symbols as in Fig. 12. Note the very narrow pronotum of *A. dytiscoides*, a distinguishing feature of this species.

***Structural features***: Body length: 7.52–8.40 mm (see Table 1). Hypomeron not visible in lateral view (as in Fig. 10C, D), lateral bead of pronotum well defined (Fig. 10D). Metasternal wing narrow, WC/WS > 3.0 (see Table 1, Figs 2A, 12). Pronotum very broad, more than or equal to 3.3× as broad as interocular distance in males (see Table 1, Figs 4D, 13).

**Figure 14.**
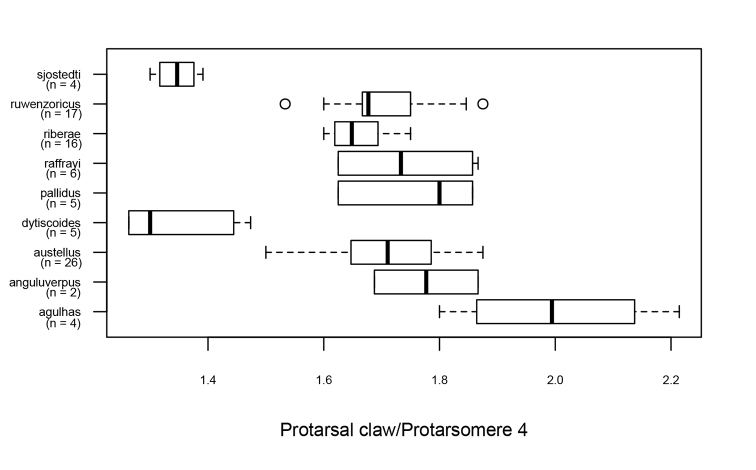
The ratio between length of protarsal claw and length of protarsomere 4 in males of the *Agabus raffrayi* group. Symbols as in Fig. 12. Note the short claws of *A. sjostedti* and *A. dytiscoides*.

***Legs***: Protarsal claws long, > 1.6× as long as protarsomere 4 (see Table 2 and Fig. 14). Metatarsomeres short and broad; metatarsomere 2 < 1.6× as long as broad (see Table 2), metatarsomere 5 < 3.0 times as long as broad (see Table 2).

***Male genitalia***: Subapically broadened, and prolonged between the subapical broadening and the apical and subapical teeth (Fig. 8E). Subapical tooth with varying appearance (similar to magnitude in variation seen in Fig. 5A–C).

**Female**: Externally similar to males.

##### Distribution.

Ethiopia (see Fig. 1).

##### Habitat.

Found in small streams and often temporary waterbodies at elevations of 2250 to 4000 m (Nilsson and Persson 1990; Nilsson 1992a; Nilsson and Persson 1993).

##### Etymology.

The name refers to the pale nature of the type specimens (Latin: *pallidus* = pale).

##### Comments.

See comments for *A. raffrayi*.

**Table 2.** Morphological characters in the *Agabus raffrayi* group. MT2L/MT2W = length/width of metatarsomere 2, MT5L/MT5W = length/width of metatarsomere 5, CL/PT4L = length of protarsal claw/length of protarsomere 4, N = number of specimens, Min = minimum value, Max = maximum value, SD = standard deviation, NA = not applicable.

Species	Sex	MT2L/MT2W					MT5L/MT5W					CL/PT4L				
		N	Min	Max	Mean	SD	N	Min	Max	Mean	SD	N	Min	Max	Mean	SD
*A. sjostedti*	♀	2	1.71	1.90	1.81	0.14	2	3.31	3.57	3.44	0.19	2	1.33	1.38	1.36	0.03
	♂	4	1.68	1.92	1.80	0.10	4	3.12	3.67	3.39	0.22	4	1.3	1.39	1.35	0.04
*A. dytiscoides*	♀	2	2.00	2.05	2.03	0.04	2	3.77	4.17	3.97	0.28	2	1.53	1.59	1.56	0.04
	♂	5	1.81	2.00	1.91	0.07	5	3.69	4.17	4.02	0.19	5	1.26	1.47	1.35	0.10
*A. anguluverpus* sp. nov.	♀	1	1.85	1.85	1.85	NA	1	3.07	3.07	3.07	NA	1	1.80	1.80	1.80	NA
	♂	2	1.86	1.95	1.90	0.07	2	3.33	3.46	3.40	0.09	2	1.69	1.87	1.78	0.13
*A. austellus* sp. nov.	♀	17	1.38	1.84	1.57	0.14	17	2.46	3.55	2.88	0.25	17	1.44	2.00	1.77	0.14
	♂	27	1.29	1.80	1.61	0.14	27	2.54	3.50	3.06	0.25	26	1.50	1.88	1.70	0.09
*A. ruwenzoricus*	♀	9	1.40	1.67	1.49	0.08	8	2.46	3.09	2.76	0.23	9	1.43	1.73	1.64	0.09
	♂	17	1.32	1.75	1.46	0.12	17	2.46	3.23	2.71	0.20	17	1.53	1.88	1.70	0.09
*A. raffrayi*	♀	5	1.31	1.43	1.37	0.05	5	2.50	2.67	2.59	0.09	5	1.46	1.93	1.69	0.18
	♂	7	1.22	1.52	1.35	0.10	6	2.31	2.92	2.54	0.21	6	1.63	1.87	1.74	0.11
*A. pallidus*	♀	6	1.30	1.50	1.37	0.09	6	2.46	2.92	2.65	0.17	6	1.67	1.86	1.77	0.07
	♂	5	1.25	1.36	1.30	0.05	5	2.36	2.67	2.48	0.12	5	1.63	1.86	1.75	0.12
*A. riberae* sp. nov.	♀	6	1.67	1.76	1.71	0.04	6	2.93	3.31	3.12	0.15	6	1.53	1.68	1.62	0.07
	♂	16	1.64	1.88	1.72	0.06	16	2.94	3.54	3.24	0.21	16	1.60	1.75	1.66	0.05
*A. agulhas* sp. nov.	♀	2	1.48	1.52	1.50	0.03	2	2.64	2.71	2.68	0.05	2	1.78	1.88	1.83	0.07
	♂	4	1.43	1.52	1.48	0.04	4	2.62	2.83	2.70	0.09	4	1.80	2.21	2.00	0.18

#### Agabus ruwenzoricus

Taxon classificationAnimaliaColeopteraDytiscidae

Guignot, 1936

2D56F90E-7240-520C-90A6-666B313140D3

Figures 1 , 4B , 8C , 11C , 11H , 12 , 13 , 14 , 15

Agabus (Agabinectes) pallidus var. ruwenzoricus Guignot, 1936: 49

##### Type locality.

“Uganda. Mons Ruwenzori, versant est, 3.000 à 4.000 m.” [Uganda, Mount Ruwenzori, eastern slope, 3000 to 4000 m].

##### Type material.

[Not examined]: Information about type specimens from Nilsson (1992a): “Lectotype here designated in NMNH (coll. Guignot) labelled: ‘Monts Ruwenzori versant est zone alpine 3000 4000 m Ch. Alluaud I 1909’, ‘3000 m’, ‘♂’, and my lectotype label; paralectotype ♂ with same original labels and my paralectotype label.”.

##### Diagnosis.

Most similar to *A. pallidus* and *A. raffrayi* but separated from these taxa by the presence of distinct interocular spots on head (compare Fig. 4B and D). The metasternal wing is rather narrow; the WC/WS frequency distribution being intermediate between *A. pallidus* and *A. raffrayi*, but most similar to *A. pallidus* (see Table 1, Fig. 12). The aedeagus has an extended portion between the subapical broadening and the apical teeth, and the pronotal hypomeron is not visible in lateral view.

##### Description.

Habitus as in Fig. 11C, H.

***Colour***: Head black with rufous anterior area; rufous interocular spots present. Pronotum black with minute to well-defined rufous margin. Elytra ferrugineous to rufopiceous. Ventral surface black, hypomeron rufotestaceous to rufous and epipleuron testaceous to rufotestaceous. Legs rufous to black. Antennae and palpi testaceous.

***Microreticulation***: Medium impressed on head, pronotum and elytra, and rather similar in both sexes. Composed of a mixture of small and somewhat larger, uneven meshes.

***Structural features***: Body length: 7.36–8.08 mm (see Table 1). Hypomeron not visible in strict lateral view (as in Fig. 10C, D, compare with 10A, B), lateral bead of pronotum narrow and well defined. Metasternal wing narrow, WC/WS 3.0 or more in most specimens (see Table 1, Fig. 12). Pronotum broad, more than twice as broad as interocular distance (see Table 1, Fig. 13).

***Legs***: Protarsal claws long, > 1.6× as long as protarsomere 4 in most specimens (see Table 2, Fig. 14). Metatarsomeres short and broad; metatarsomere 2 < 1.8× as long as broad (see Table 2), metatarsomere 5 < 3.3× as long as broad (see Table 2).

***Male genitalia***: Subapically broadened and prolonged between the subapical broadening and the apical and subapical teeth (Fig. 8C). Subapical tooth with varying appearance (similar to magnitude in variation seen in Fig. 5A–C).

**Female**: Externally similar to males.

##### Distribution.

Kenya, Rwanda, Uganda and the Democratic Republic of the Congo. Nilsson (1992a) and Omer-Cooper (1965) also give South Africa and Zimbabwe but these records are likely to belong to other species (see below).

##### Habitat.

**Figure 15.**
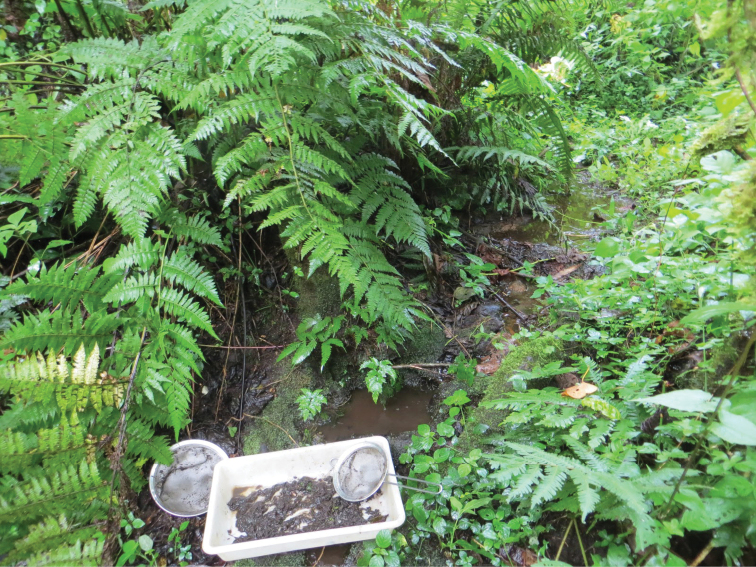
Small forest stream at 1900 m in remaining fragment of afromontane cloud forest on Vuria, the highest mountain in the Taita Hills, Taita-Taveta district, northeastern part of Eastern Arc Mountains, Kenya. Albeit minute, the small pools seen in the photo were inhabited by tens of individuals of *Agabus ruwenzoricus*. January 19, 2016.

Most records are from small mountain streams and rivers at elevations of 1900 to 3100 m, but it has also been found in stagnant waterbodies (Nilsson 1992a). We found the species in a very small cold-water forest stream at an elevation of 1900 m in the Taita Hills, Kenya (Fig. 15).

##### Etymology.

The name refers to the locality where the species was first collected, Mount Ruwenzori.

#### Agabus sjostedti

Taxon classificationAnimaliaColeopteraDytiscidae

Régimbart, 1908

CA2A49ED-3A9F-5F34-886E-70D6F97B8E34

Figures 1 , 4C , 8A , 10A , 11A , 11F , 12 , 13 , 14

Agabus sjostedti Régimbart, 1908: 5–6

##### Type locality.

“mont Meru, entre 3,500 et 4,300 mèt” [Tanzania, Mount Meru, between 3500 and 4300 m].

##### Type material.

***Lectotype*** ♂ (NHRS) labelled: “Meru Regenwald”, “Meru Sjöstedt”, “Type”, “Typus”, “Agabus sjöstedti Rég. n.sp. ♂ et ♀ “LECTOTYPUS ♂ Agabus sjostedtiRégimbart 1908, Des. A.Nilsson -90”. Paralectotypes 2 ♂ 1 ♀ (NHRS) labelled: “Meru Regenwald”, “Meru Sjöstedt”, “22 dec”, “PARALECTOTYPUS ♂/♀ Agabus sjostedtiRégimbart 1908 Des. A.Nilsson 1990”.

##### Diagnosis.

Most similar to *A. dytiscoides* but separated by its larger size (see Table 1), narrower metasternal wing (see Table 1), broader prontoum (see Table 1 and compare Fig. 4C with Fig. 4A) and by the curvature of the aedeagal subapical tooth (compare Fig. 8A with Fig. 8B). The aedeagus is prolonged preapically as in the preceeding three species, but the pronotal hypomeron is clearly visible in strict lateral view (see Fig. 10A).

##### Description.

Habitus as in Fig. 11A, F.

***Colour***: Head black to rufous with testaceous to rufous interocular spots. Pronotum rufopiceous to black and rufous to testaceous at margins; some specimens with two diffuse rufous to testaceous spots in the middle of the pronotum. Elytra ferruginous to rufopiceous. Ventral surface rufopiceous to black, hypomeron and epipleuron testaceous. Legs rufous to rufopiceous. Antennae and palpi testaceous to rufous.

***Microreticulation***: Males with medium impressed reticulation on head and pronotum and slightly finer reticulation on elytra giving a shiny appearance, all meshes being a mix of smaller and somewhat larger meshes.

The two females studied varied greatly in microreticulation, but shared having mostly isodiametric meshes on elytra and the same variable meshes on head and pronotum as males. One female (Mt. Meru) had very coarse meshes; giving head, pronotum and elytra a matte appearance while the other female (Kilimanjaro Bismarck hut) had the same shiny appearance as males.

***Structural features***: Body length: 8.08–9.12 mm (see Table 1). Hypomeron broadly visible in strict lateral view (see Fig. 10A), lateral bead of pronotum broad and well defined (see Fig. 10A). Metasternal wing very narrow, WC/WS > 3.6 in most specimens (see Table 1, Fig. 12). Pronotum broad, more than twice as broad as interocular distance (see Table 1, Figs 5C, 13).

***Legs***: Protarsal claws short, < 1.4× as long as protarsomere 4 (see Table 2, as in Figs 3A, 14). Metatarsomeres long and slender; metatarsomere 2 > 1.6× as long as broad (see Table 2), metatarsomere 5 > 3.3× as long as broad in most specimens (see Table 2).

***Male genitalia***: Subapically broadened, and prolonged between the subapical broadening and the apical and subapical teeth. Subapical tooth robust, with distinct curvature (see Fig. 8A).

**Female**: Elytral and pronotal microreticulation much coarser than in males.

##### Distribution.

Known from Meru and Kilimanjaro mountains in northern Tanzania (see Fig. 1).

##### Habitat.

Régimbart (1908) reports that the type specimens (from Mt. Meru) were found in very cold water, at an altitude of 3500 to 4300 m. On Mt. Kilimanjaro it has been found at lower altitudes between 2200 and 3100 m (Nilsson 1992a).

##### Etymology.

The name refers to the collector of the type specimens, Yngve Sjöstedt.

##### Comments.

Nilsson (1992a) studied the material collected by G.F. De Witte and concluded that the animals that Gschwendtner (1938) and Guignot (1959) referred to as *Gaurodytes sjostedti* from Park National Albert [=Virunga NP in DRC], bordering the Ruwenzori mountains, were in fact *A. ruwenzoricus*. Older records of *A. sjostedti* must be interpreted with caution.

#### Agabus dytiscoides

Taxon classificationAnimaliaColeopteraDytiscidae

Régimbart, 1908

51819432-1077-567E-AD2A-0AEAD2758C40

Figures 1 , 3A , 4A , 8B , 10B , 11B , 11G , 12 , 13 , 14

Agabus dytiscoides Régimbart, 1908: 6–7

##### Type locality.

“Kiboscho, au Kilimandjaro … entre 3,000 et 3,500 mèt.” [Tanzania, Kiboscho, Mount Kilimanjaro, between 3000 and 3500 meters].

##### Type material.

***Lectotype*** ♂ (NHRS) labelled: “Kilimandj. Sjöstedt”, “Kiboscho 3’ -4000 m.”, “20 febr”, “LECTOTYPUS ♂ Agabus dytiscoidesRégimbart 1908 Des. Nilsson 1990”. Paralectotypes 5 ♂ 3 ♀ (NHRS) labelled: “Kilimandj. Sjöstedt”, “Kiboscho 3’ -4000 m.”, “20 febr”, “PARALECTOTYPUS ♂/♀ Agabus dytiscoidesRégimbart 1908 Des. Nilsson 1990”.

##### Diagnosis.

The very narrow pronotum, distinctly narrower than the base of elytra, is characteristic and separates *A. dytiscoides* from all other species in the group (see Table 1, Fig. 4A). With the pronotal hypomeron clearly visible in strict lateral view (see Fig. 10B), *A. dytiscoides* is most similar to *A. sjostedti* but can be separated, apart from the pronotal shape, by its smaller size (see Table 1), broader metasternal wing (see Table 1), and by the shape of the less robust aedeagal subapical tooth (compare Fig. 8A, B).

##### Description.

Habitus as in Fig. 11B, G.

***Colour***: Head rufopiceous to black with testaceous to rufous interocular spots. Pronotum rufopiceous to black with testaceous margins; some specimens with two diffuse rufous to testaceous spots in the middle of the pronotum. Elytra ferruginous to brown. Ventral surface rufopiceous to black, hypomeron and epipleuron testaceous. Legs rufous. Antennae and palpi testaceous.

***Microreticulation***: Males with medium impressed reticulation on head and pronotum. Females with much coarser meshes than males, giving pronotum and elytra a matte appearance. Females also with mostly elongate meshes on pronotum. Males with a mixture of small and somewhat larger meshes on both pronotum and elytra, while female elytra tends to have more uniform small meshes. Both sexes with overall larger meshes on pronotum than elytra.

***Structural features***: Body length: 7.36–7.76 mm (see Table 1). Pronotal hypomeron broadly visible in strict lateral view (see Fig. 10B), lateral bead of pronotum well defined. Metasternal wing relatively broad, WC/WS < 3.6 (see Table 1, Fig. 12). Pronotum very narrow, < 2.0× as broad as interocular distance (see Table 1, Figs 4A, 13), clearly narrower than base of elytra and therefore with a non-continuous outline between pronotum and elytra.

***Legs***: Protarsal claws short, < 1.6× as long as protarsomere 4 (see Table 2, Figs 3A, 14). Metatarsomeres very long and slender; metatarsomere 2 > 1.8× as long as broad (see Table 2), metatarsomere 5 > 3.6× as long as broad (see Table 2).

***Male genitalia***: Subapically broadened, and prolonged between the subapical broadening and the apical and subapical teeth. Subapical tooth with curvature as in Fig. 8B, less robust than in *A. sjostedti*.

**Female**: Elytral and pronotal microreticulation much coarser than in males.

##### Distribution.

Known from Kilimanjaro and the Loolmalasin mountains in northern Tanzania (see Fig. 1).

##### Habitat.

Régimbart (1908) reports that the type specimens were found in cold runoff water from a glacier at 3000 to 3500 m.

##### Etymology.

The name literally translates to “*Dytiscus*-like”. In his original description Régimbart (1908) explains that “J’ai donné à l’*A. dytiscoides* ce nom à cause de la grande similitude de forme et de couleur des males dans les deux especes. [I gave it the name *A. dytiscoides* because of the great similarity in form and colour between the males in the two species.]”. Despite great differences in size and many other characters, the pale pronotal margins and the distinct shoulder between the pronotum and elytra are somewhat reminiscent of a *Dytiscus*. That said, Régimbart also mentioned similarities with *A. raffrayi*, making it difficult to be sure exactly what he was referring to in choosing this name.

#### Agabus anguluverpus

Taxon classificationAnimaliaColeopteraDytiscidae

Englund, Njoroge & Bergsten sp. nov.

5BE56490-C906-5CB9-BC78-B41064D08DA3

http://zoobank.org/78EDD21C-BB25-4472-872C-6159BBAD12E8

Figures 1 , 7A , 8F , 10C , 11K , 11O , 12 , 13 , 14 , 20 , 21

##### Type locality.

Kenya, Mount Kenya, Chogoria, Lake Ellis, -0.123N, 37.401E.

##### Type material.

***Holotype*** ♂ (NMK) labelled: “Kenya, Mt. Kenya, Lake Ellis, Chogoria. -0.123S 37.401E. 17.IX.2015 Leg. W. Wamiti”. ***Paratypes*** 1 ♂ 1 ♀ (NHRS, NMK) labelled: “Kenya, Mt. Kenya, Lake Ellis, Chogoria. -0.123S, 37.401E. 17.IX.2015 Leg. W. Wamiti”.

##### Diagnosis.

This species is in some respects similar to *A. sjostedti* and *A. dytiscoides* in that females are matte due to a coarse dorsal microsculpture, and although the pronotal hypomeron is not or barely visible in strict lateral view, the pronotal bead is broader anteriorly (see Fig. 10C). The subapical portion of aedeagus is not prolonged, which is similar to South African species of the group, but the apex is straight in ventral view which is unique in the group. The aedeagus is evenly thickened, and essentially lacks the subapical broadening seen in most *raffrayi* group taxa (see Fig. 8).

##### Description.

Habitus as in Fig. 11K, O.

***Colour***: Head rufopiceous with testaceous interocular spots and an anterior testaceous area. Pronotum brown to rufopiceous with testaceous margins; some specimens with a diffuse rufotestaceous area in the middle of the pronotum. Elytron brown to testaceous brown. Ventral surface rufous, hypomeron testaceous, epipleuron testaceous brown. Legs rufous to testaceous. Antennae and palpi testaceous. The three specimens collected were all teneral individuals, especially the two paratypes, and as a result there is a probability that non-teneral individuals of this species will be somewhat darker than described here. In particular the pronotum may be darker medially in non-teneral individuals.

***Microreticulation***: Males with medium impressed reticulation on head and pronotum. Females with much coarser and larger meshes than males, giving pronotum and elytra a matte appearance. Both sexes with a mixture of small and somewhat larger meshes.

***Structural features***: Body length: 7.36–7.52 mm (see Table 1). Hypomeron not or barely visible in strict lateral view (see Fig. 10C), lateral bead of pronotum broad and well defined, broader anteriorly (see Fig. 10C). Metasternal wing narrow, WC/WS 3.0 or more in both males and females (see Table 1 and Fig. 12). Pronotum more than twice as broad as interocular distance (see Table 1 and Fig. 13), lateral margins straighter anteriorly and more curved posteriorly.

***Legs***: Protarsal claws long, > 1.6× as long as protarsomere 4 (see Table 2, Fig. 14). Metatarsomeres very long and slender; metatarsomere 2 > 1.8× as long as broad in both females and males (see Table 2), and metatarsomere 5 > 3.3× as long as broad in males (see Table 2).

***Male genitalia***: Aedeagus without distinct subapical broadening and without subapical prolongation; subapical tooth angled both basally and subapically (see Fig. 8F). Aedeagus with straight apex in ventral view.

**Female**: Elytral and pronotal microreticulation much coarser than in males.

##### Distribution.

Only known from Mount Kenya in central Kenya (see Fig. 1).

##### Habitat.

Lake Ellis is situated at an altitude of about 3500 m on Mount Kenya’s eastern slope (Figs 20, 21).

##### Etymology.

The species name refers to the angled subapical tooth of the male genitalia (Latin: *angulus* = angle, *verpus* = penis).

#### Agabus austellus

Taxon classificationAnimaliaColeopteraDytiscidae

Englund, Bilton & Bergsten sp. nov.

C27284B6-6C84-5A1D-BF4A-869F9F3805C0

http://zoobank.org/290F05EF-3F4E-4971-B957-1071B64FBD13

Figures 1 , 5D , 6A–D , 7B , 8G , 9A–D , 11L , 11P , 12 , 13 , 14 , 16 , 17

##### Type locality.

**Figure 16.**
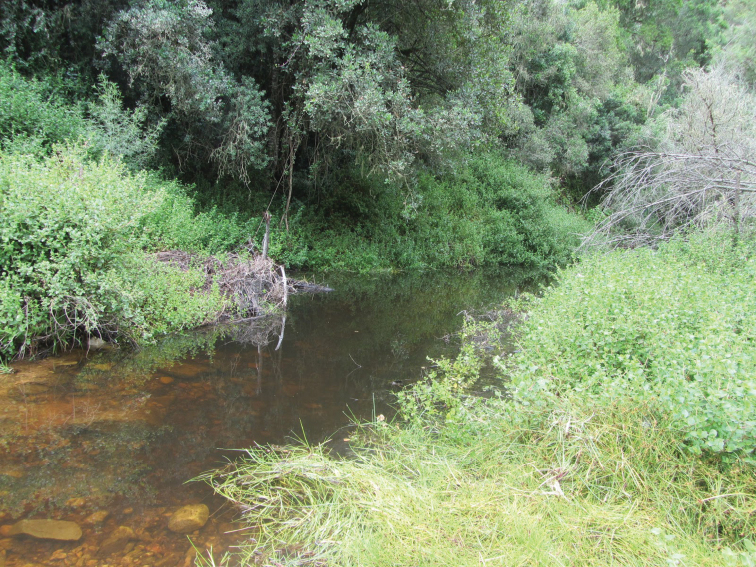
Diep River at 250 m, tributary to Keurbooms River, R339 towards Prince Alfred’s Pass, Langkloof Mountains, Western Cape province, South Africa. Type locality of *Agabus austellus* sp. nov. December 13, 2015.

South Africa, Western Cape Province, Tributary stream to Keurboom river crossing R339 road, 33.8612S, 23.1729E, 250 m (Fig. 16).

##### Type material.

***Holotype*** ♂ (AMG) labelled: “South Africa W Cape Province Tributary stream to Keurboom river x-ing R339. 33.8612S, 23.1729E, 250m. Foreststream with gravel and veg. At edges RSA15-21 13.XII.2015 Leg. J. Bergsten & A. Désamoré”. ***Paratypes***: 1 ♂ (NHRS) labelled: “South Africa E Cape Province. Small fish river x-ing road r337, NW of Somerset East. 32.5913S, 25.4510E, 1017m. Waterpools in streambed RSA15-27 15.XII.2015 Leg. J. Bergsten & A. Désamoré”; paratypes 1 ♂ (NHRS) labelled: “Rep. South Africa, W. Cape Gydo Pass at junc R303 & Witzenberg Valley Rd. Aug. 25, 2004 G. Challet”; paratypes 6 ♂ 2 ♀ (MZLU) labelled: “S. Afr. Cape Prov., Swartbergpas, Platberg, Alt.: ab. 5000 ft., 5–6.I.51 No. 120”, “Swedish South Africa Expedition 1950–1951, Brinck-Rudebeck”, “Agabus ruwenzoricus Guignot, det AN Nilsson 1990”, one male and one female had an extra label containing “Agabus raffrayi sharp, Det. J. Omer-Cooper”; paratypes 2 ♂ 3 ♀ (MfN) labelled: “R.S.Africa 17.xi.1997 32°15.3'S, 22°29.9'E Cape Province:Karoo NP. Swamp at Puttersvlei, swamp vegetation treating, watercatcher, lg. M.Uhlig+J.Ndamane”, one male had an extra label containing “Agabus ruwenzoricus Guignot det. K.B. Miller 1999”; paratypes 2 ♂ 3 ♀ (AMG, CBP) labelled: “Sept. 2002 South Africa WC Pools beside Brée River below Mitchell’s Pass, Ceres. D T Bilton leg.”; paratypes 4 ♂ 1 ♀ (CBP, ZSM) labelled: “24/ix/2009 South Africa WC Groote Swartberg stream on R328 1 km N of De Top, on N side of pass. D.T. Bilton leg.”; paratypes 1 ♂ 1 ♀ (CBP) labelled: “20/ix/2014 South Africa WC Gifberg – stream in Gifberg Pass above Vanrhynsdorp rocky stream. D T Bilton leg.”; paratype 1 ♂ (CBP) labelled: “11/ii/2017 South Africa WC Harkerville Forest pool 1 survey site 16. M Bird & D T Bilton leg.”; paratypes 6 ♂ 6 ♀ dry mounted, 18 ♂ 16 ♀ in ethanol (AMG, CBP, NHRS) labelled: “28/v/2018 South Africa KZN Drakensberg – river nr. Sentinel Peak 28°45'30.80"S, 28°54'14.82"E 2960m M. Mlambo leg.”; paratype 1 ♀ (IBE) labelled: “25/viii/2008 South Africa WC Gydo Pass – pool at Jnct. R303 & Witzenberg Valley Rd. G Challet leg.”; paratypes 1 ♂ (IBE) labelled: “2/x/2010 South Africa WC Cederberg Dwarsrivier 32°30'59.51"S, 19°21'E 735 m Hidalgo-Galiana & Kleynhans leg.”; paratypes 1 ♂ (CBP) labelled: “6/x/2015 South Africa NC Bokkeveld, Avontuur – stream below Fynbos Cottage D T Bilton leg.”; paratypes 4 ♂ 3 ♀ (CBP, ISAM, SANC) labelled: “22/ix/2010 South Africa WC Gydo Pass stream along Witzenberg Valley road ca 1 km SW of Jnct. with R303 985 m D T Bilton leg.”.

##### Diagnosis.

Most similar to *A. riberae* sp. nov. and *A. agulhas* sp. nov., but distinguishable by a combination of having a scutellum darker than or as dark as elytra, base of aedeagal subapical tooth lacking a distinct incurvation (compare Fig. 8G, I) and a relatively narrow metasternal wing (see Table 1). The pronotal hypomeron is not visible in strict lateral view, the aedeagus does not have a prolonged subapical portion and in ventral view its apex is asymmetrically curved. The discal elytral microreticulation of most specimens is dominated by relatively small, isodiametric meshes.

##### Description.

Habitus as in Fig. 11L, P.

***Colour***: Head black, with rufous interocular spots; some specimens with an additional anterior rufous area. Pronotum rufopiceous to black. Elytra rufopiceous to black. Ventral surface rufous to black; testaceous lines on abdominal segments rarely present; hypomeron and epipleuron rufopiceous to rufous. Legs rufous to rufopiceous. Antennae and palpi testaceous.

***Microreticulation***: Relatively fine on both pronotum and elytra, and rather similarly impressed in both sexes. The microreticulation of the elytral disc is dominated by relatively small, somewhat isodiametric meshes in most specimens examined (e.g. Fig. 9B, C), although this character does vary somewhat between populations in this relatively widespread species (see Fig. 9). In particular, the male from Harkerville Forest (see Fig. 9D) has a reticulation composed of much larger meshes than seen in other material of this species. This specimen conforms to *A. austellus* sp. nov. on other morphological characters, and COI sequence data (I. Ribera, pers. comm.).

***Structural features***: Body length: 6.80–8.40 mm (see Table 1). Hypomeron marginally visible in strict lateral view, lateral bead of pronotum narrow and well defined. Metasternal wing narrow, WC/WS 3.1 or more in both males and females (see Table 1 and Fig. 12). Pronotum broad, more than twice as broad as interocular distance (see Table 1 and Fig. 13).

***Legs***: Protarsal claws long, > 1.6× as long as protarsomere 4 in most specimens (see Table 2 & Fig. 14). Metatarsomeres usually short and broad; metatarsomere 2 < 1.8× as long as broad in most specimens (see Table 2), metatarsomere 5 < 3.3× as long as broad in most specimens (see Table 2).

***Male genitalia***: Aedeagus lack the prolonged section between the subapical broadening and the apical and subapical teeth present in some species in the group (see Fig. 8G). In ventral view the apex is asymmetrically curved (Fig. 7B). There is some variation in the shape and size of the subapical tooth of the aedeagus (Fig. 6A–D), this being relatively small in most populations (Fig. 6A–C).

**Female**: Externally similar to males. Some specimens with dorsal microreticulation slightly more strongly impressed.

##### Distribution.

Republic of South Africa, where the species is relatively widespread, from the Bokkeveld Plateau in the south of the Northern Cape Province, most mountain systems of the Western Cape Province and east along the Great Escarpment to the Drakensberg (see Fig. 1). This wide geographical range encompasses winter, summer and bimodal rainfall regimes.

##### Ecology.

**Figure 17.**
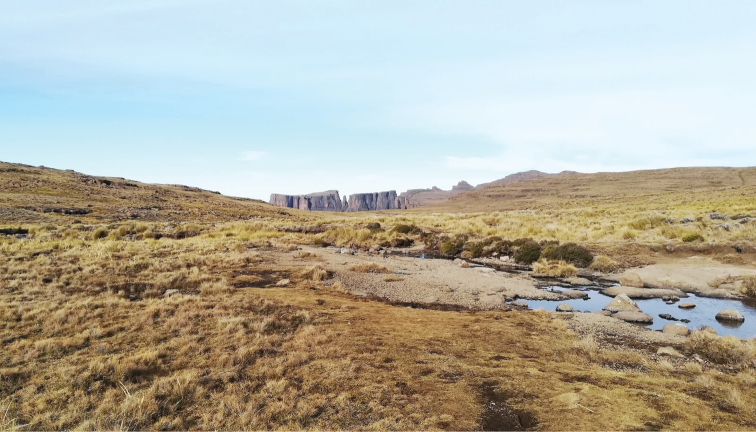
River nr Sentinel Peak, 2960 m, Drakensberg, KwaZulu-Natal, South Africa. Locality for *Agabus austellus* sp. nov. May 28, 2018. Photo Samuel Motitsoe.

Found in streams, pools beside streams and remnant pools in seasonal running watercourses. Most localities are situated in Fynbos or alpine grassland (e.g., Fig. 17), but also recorded from densely forested streams. Sites span a wide range of altitudes, from the type locality at 250 m (Fig. 16) to almost 3000 m in the Kwazulu-Natal, Drakensberg (Fig. 17), most being at intermediate elevations.

##### Etymology.

The name refers to the fact that the species is widespread in South Africa and therefore truly an *Agabus* of the south (Latin: *austellus* = southern parts).

##### Comments.

Nilsson (1992a) and Omer-Cooper (1965) assigned specimens from South Africa and S. Rhodesia [=Zimbabwe] to *A. ruwenzoricus*. We have studied one series of specimens cited by both authors from the Swartberg Pass (Swartbergpas) W. Cape Province, housed in Lund (MZLU), which correspond to *A. austellus* sp. nov. Males of this species do not have a prolonged preapical section of the aedeagus, a distinguishing character not previously noted in the *raffrayi* group. It seems likely that all previous records of *A. raffrayi*, *A. pallidus* or *A. ruwenzoricus* from South Africa, and also possibly those from Zimbabwe, are misidentified and mostly refer to *A. austellus* sp. nov. This species is somewhat variable in body shape, elytral microreticulation (see Fig. 9A–D) and the shape and size of the subapical tooth of the aedeagus (see Fig. 6A–D). We interpret this variation as comprising a single species, however, particularly given the very similar mtDNA COI sequences observed amongst populations spanning the entire known range in South Africa (i.e., Bokkeveld Plateau to Drakensberg, I. Ribera, pers. comm.).

#### Agabus riberae

Taxon classificationAnimaliaColeopteraDytiscidae

Bilton, Englund & Bergsten sp. nov.

34A110FC-1AAA-506E-AD44-515DBF8E5B46

http://zoobank.org/A391EC84-95B1-480F-B4DD-E7DEA2AE3076

Figures 1 , 6E, F , 8H , 9E , 11M , 11Q , 12 , 13 , 14 , 18 , 19

##### Type locality.

South Africa, Northern Cape Province, Kamiesberg, stream on Witwater-Langkloof Road ca 1 km S. of junction, 30°23'41.30"S 18°08'07.95"E, 1100 m.

##### Type material.

***Holotype*** ♂ (AMG) labelled: “19/ix/2010 South Africa NC Kamiesberg Stream on Witwater-Langkloof road ca 1 km S. of junction. D.T. Bilton leg.”. ***Paratypes***: 5 ♂ 5 ♀ (CBP, SANC, ZSM) labelled: “19/ix/2010 South Africa NC Kamiesberg Stream on Witwater-Langkloof road ca 1 km S. of junction. D.T. Bilton leg.”; paratype 4 ♂ 4 ♀ (AMG, CBP, ISAM, NHRS) labelled: “19/1x/2010 South Africa NC Kamiesberg – stream above Studer Pass ca 5 km W of Witwater 30°23'13.49"S, 18°07'05.78"E 1,105 m D T Bilton leg.”; paratype 1 ♀ (IBE) labelled: “22/viii/2004 South Africa NC Kamiesberg – stream at top of Studer Pass G. Challet leg.”; paratype ♂ (CBP) labelled: “19/ix/2014 South Africa NC Kamiesberg temporary stream in Kamiesberg Pass D T Bilton leg.”; paratypes 2 ♂ (CBP) labelled: “18/ix/2014 South Africa NC Kamiesberg spring pool on Rondefontein Farm 30°30'05.59"S, 18°08'56.35"E 1023 m D T Bilton leg.”; paratypes 4 ♂ 1 ♀ (CBP, NHRS) labelled: “17/ix/2014 South Africa NC Kamiesberg stream nr. Damsland on N. side of Rooiberg 30°23'36.33"S, 18°06'32.12"E 1111 m D T Bilton leg.”; paratype 1 ♂ (CBP) labelled: 19/ix/2014 South Africa NC Kamiesberg stream nr. De Kuilen 30°10'44.94"S, 18°04'37.71"E 940 m D T Bilton leg.”; paratypes 2 ♂ (CBP) labelled: “18/ix/2014 South Africa NC Kamiesberg – stream at bottom of Langkloof 30°33'16.98"S, 18°08'19.13"E 594 m D T Bilton leg.”; paratypes 2 ♂ 4 ♀ (AMG, CBP) labelled: “28/ix/2018 South Africa WC Cederberg Tra-Tra river @ Wupperthal 32°16'45.35"S, 19°13'04.32"E 485 m D T Bilton leg.”.

##### Diagnosis.

Very similar to *A. austellus* sp. nov., differing from this species in having a relatively broad metasternal wing (see Table 1), and an elytral microreticulation dominated by larger, more irregular meshes than seen in most *A. austellus* sp. nov. specimens (see above). Metatarsomere 5 is also somewhat longer in this species than in most *A. austellus* sp. nov.

##### Description.

Habitus as in Fig. 11M, Q.

***Colour***: Head black with rufous interocular spots and an anterior rufous area. Pronotum black with rufous borders. Elytra rufopiceous to black. Ventral surface black, testaceous lines on abdominal segments rarely present, hypomeron and epipleuron rufotestaceous to rufous. Legs rufous to rufopiceous. Antennae and palpi testaceous.

***Microreticulation***: Relatively fine on both pronotum and elytra, and rather similarly impressed in both sexes. The microreticulation of the elytral disc is typically dominated by relatively large, somewhat irregular meshes (Fig. 9E).

***Structural features***: Body length: 7.21–8.24 mm (see Table 1). Hypomeron marginally visible in strict lateral view, lateral bead of pronotum narrow and well defined. Metasternal wing broad, WC/WS 3.0 or less in most specimens (see Table 1 and Fig. 12). Pronotum broad, more than twice as broad as interocular distance (see Table 1 and Fig. 13).

***Legs***: Male protarsal claws long, > 1.6× as long as protarsomere 4 (see Table 2 and Fig. 14). Metatarsomere 2 short and broad, < 1.8× as long as broad (see Table 2) in most specimens. Metatarsomere 5 long and slender, > 3.3× as long as broad in most males (see Table 2).

***Male genitalia***: Tip of aedeagus short, lacking the prolongation of the area located between the subapical broadening and the apical and subapical tooth present in some species in the group (see Fig. 8H). There is some variation in the shape and size of the subapical tooth (Fig. 6E, F), this being relatively long and narrow in most specimens examined (Fig. 6F), but with both narrow and broader teeth being observed within the same population.

**Female**: Externally similar to males. Some specimens with dorsal microreticulation slightly more strongly impressed.

##### Distribution.

To date known only from Kamiesberg Range in the Northern Cape Province, and the eastern fringes of the Cederberg, Western Cape Province, Republic of South Africa (see Fig. 1), material from both areas being confirmed from COI sequences. The Kamiesberg represents a northerly outlier of Fynbos and Renosterveld vegetation in predominantly arid Namaqualand, and consequently have a diverse flora with a number of localised endemics (Helme and Desmet 2006). The mountains support the bulk of the global population of the endemic dytiscid *Andex insignis* Sharp, 1882 and a number of new, apparently endemic, water beetles have been described from the area in recent years (Bilton 2013, 2015, 2016). *Agabus riberae* sp. nov. appears to be the only *Agabus* present in Kamiesberg, where it is abundant. In the Cederberg the species has been found close to Wupperthal, in the relatively dry northeastern fringes of the range. *A. austellus* sp. nov. is the only species so far recorded from the wetter central areas of the Cederberg. All sites known to date experience predominantly winter rainfall.

##### Ecology.

**Figure 18.**
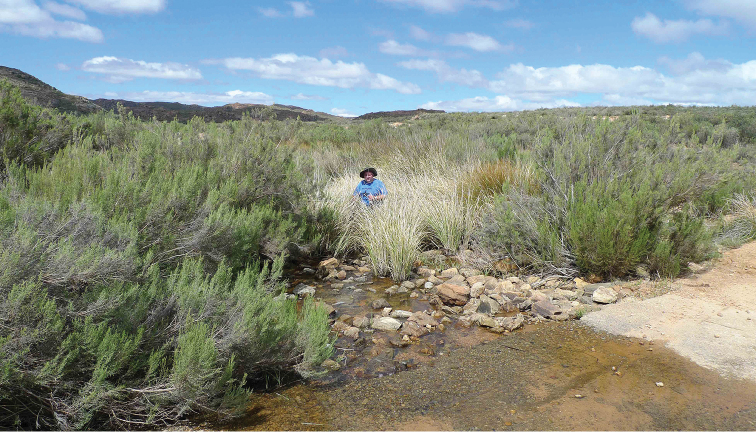
Stream at 1100 m on Witwater-Langkloof road ca 1 km S. of junction, Kamiesberg, Northern Cape Province, South Africa. Type locality of *Agabus riberae* sp. nov., with DTB. September 19, 2010. Photo Rebecca Bilton.

**Figure 19.**
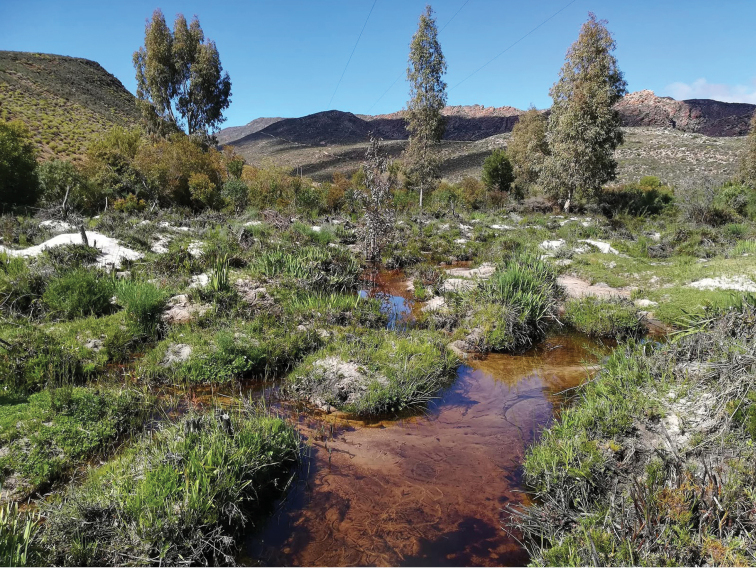
Tra-Tra River at 485 m at Wupperthal, Cederberg range, Western Cape Province, South Africa. Locality for *Agabus riberae* sp. nov. September 28, 2018. Photo Stacey DeAmicis.

Found in streams and associated pools in the Kamiesberg and northeastern Cederberg ranges (Figs 18, 19), in either Fynbos or Renosterveld vegetation between 480 and 1000 m elevation. Typically netted from marginal vegetation, including at the base of tussocks. Also found amongst grasses in a spring pool with cold water. Ecological differences between this species and *A. austellus* sp. nov. are unclear, but may relate, at least in part, to rainfall.

##### Etymology.

Named after our late friend and colleague Ignacio Ribera, who will be sorely missed.

##### Comments.

Despite the relatively minor morphological differences between this species and *A. austellus* sp. nov. we consider these two taxa distinct. COI sequences for four specimens of *A. riberae* sp. nov. investigated differed by 4.5–4.7% from *A. austellus* sp. nov., more than that observed between many well-established species in the subgenus Acatodes Thomson, 1859 (I. Ribera pers. comm.). This is supportive of the recognition as a distinct taxon, particularly given the relative uniformity in COI sequence observed across the wide geographical range of *A. austellus* sp. nov.

#### Agabus agulhas

Taxon classificationAnimaliaColeopteraDytiscidae

Bilton, Englund & Bergsten sp. nov.

49160C6A-2226-5941-BC5E-7342B0895666

http://zoobank.org/FD6B9312-065E-4B96-9FDF-9B1C45EA8D69

Figures 1 , 6G , 8I , 9F , 11N , 11R , 12 , 13 , 14

##### Type locality.

South Africa, Western Cape Province, Rooistrandveld, Bredasdorp, natural viei beside road to Die Dam at Ratelrivier 34°43'00.47"S, 19°41'53.81"E, 31 m.

##### Type material.

***Holotype*** ♂ (AMG) labelled: “26/ix/2010 South Africa WC Rooistrandveld, Bredasdorp natural viei beside road to Die Dam @ Ratelrivier FW marsh with tussocks etc. D. T. Bilton leg.”. ***Paratypes*** 3 ♂ 2 ♀ (AMG, CBP, NHRS, ZSM) labelled: “26/ix/2010 South Africa WC Rooistrandveld, Bredasdorp natural viei beside road to Die Dam @ Ratelrivier FW marsh with tussocks etc. D. T. Bilton leg.”.

##### Diagnosis.

Very similar to *A. austellus* sp. nov. and *A. riberae* sp. nov., but distinguishable by the distinctly curved base of the aedeagal subapical tooth (compare Fig. 8I with Fig. 8G, H and see Fig. 6G), the scutellum being lighter than the elytra and its relatively narrow metasternal wing (see Table 1 and Fig. 12).

##### Description.

Habitus as in Fig. 11N, R.

***Colour***: Head black with weak rufous interocular spots and an anterior rufous area. Pronotum black with slightly rufous margins. Elytra blackish brown to black, with a lighter scutellum. Ventral surface black, testaceous lines on abdominal segments reduced or absent, hypomeron and epipleuron rufotestaceous to rufous. Legs rufopiceous to rufous. Antennae and palpi testacous.

***Microreticulation***: Relatively fine on both pronotum and elytra, and slightly more impressed in females. The microreticulation of the elytral disc is composed of a mix of small and larger, somewhat irregular meshes (Fig. 9F).

***Structural features***: Body length: 7.60–8.00 mm (see Table 1). Hypomeron marginally visible in strict lateral view, lateral bead of pronotum narrow and well defined. Metasternal wing very narrow, WC/WS > 3.6 in all specimens (see Table 1 and Fig. 12). Pronotum broad, more than twice as broad as interocular distance (see Table 1 and Fig. 13).

***Legs***: Protarsal claws very long, > 1.8× as long as protarsomere 4 in all males (see Table 2 & Fig. 14). Metatarsomeres short and broad; metatarsomere 2 < 1.8× as long as broad (see Table 2), metatarsomere 5 < 3× as long as broad in all specimens (see Table 2).

***Male genitalia***: Aedeagus without the prolonged section between subapical broadening and the apical and subapical teeth which is present in some species in the group. In ventral view the apex is asymmetrically curved. Base of subapical tooth distinctly curved basally (see Figs 8I, 6G).

**Female**: Externally similar to males. Dorsal microreticulation slightly more impressed than in males.

##### Distribution.

Only known from the type locality, a lowland valley wetland at 31 m on the Agulhas Plain, Western Cape Province, Republic of South Africa (see Fig. 1). The most southerly distributed *Agabus* species in the world.

##### Ecology.

Collected from the base of large tussocks in a valley wetland. Despite having largely lentic conditions, this is likely to experience some seepage flow, particularly following periods of high rainfall in winter and spring.

##### Etymology.

Named after the Agulhas Plain, on which the type locality is situated. The Agulhas Plain is itself named in reference to nearby Cape Agulhas (Portuguese – Cabo das Agulhas = Cape of Needles), the most southerly point on the African continent. As with other members of the species group, *A. agulhas* sp. nov. has sharp, needle-like, teeth at the aedeagal apex.

##### Comments.

COI sequence divergence between *A. agulhas* sp. nov. and *A. austellus* sp. nov. ranges from 3.9 to 4.7%; that between *A. agulhas* sp. nov. and *A. riberae* sp. nov. being 6.4% (I. Ribera, pers. comm.).

**Figure 20.**
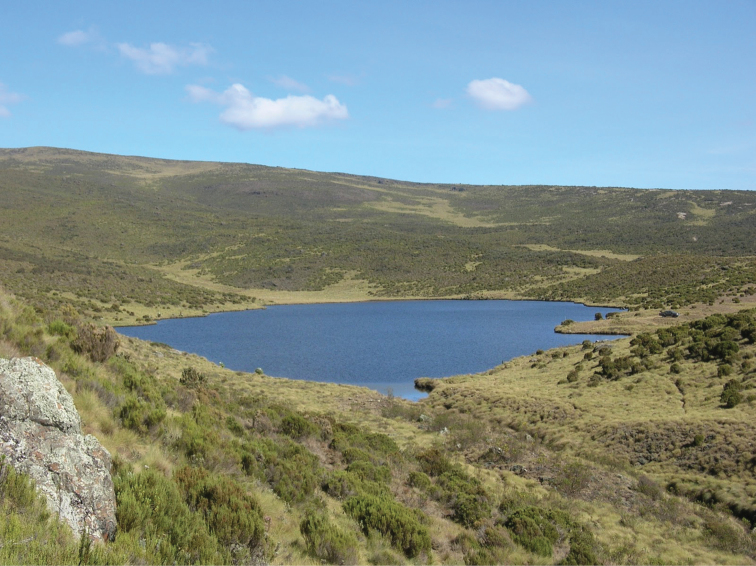
Lake Ellis on Mount Kenya, Kenya. Type locality for *Agabus anguluverpus* sp. nov. September 17, 2015. Photo Wanyoike Wamiti.

**Figure 21.**
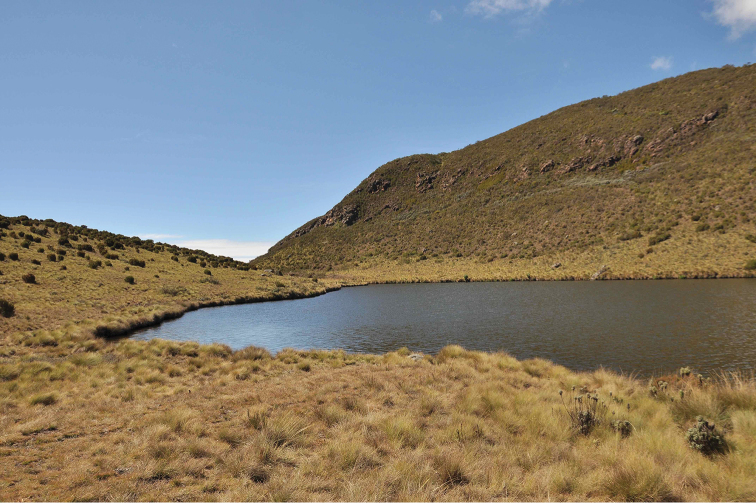
Part of Lake Ellis on Mount Kenya, Kenya. Type locality for *Agabus anguluverpus* sp. nov. September 17, 2015. Photo Wanyoike Wamiti.

## Discussion

*Agabus* is one of a number of largely temperate northern hemisphere water beetle lineages which have colonised the Afrotropical region. Other examples include *Nebrioporus* Régimbart, 1906 and *Ilybiosoma* Crotch, 1873 within the Dytiscidae and *Helophorus* Fabricius, 1775 (Hydrophiloidea, Helophoridae). In all cases, these genera are restricted to relatively high elevations in East Africa, from Ethiopia southwards, but occur across a much greater range of altitudes in temperate regions of South Africa, particularly the Cape. South African *Agabus* have to date been considered to belong to *Agabus ruwenzoricus* (Nilsson 1992a), or prior to the recognition of *A. ruwenzoricus*, *Agabus pallidus* (Omer-Cooper 1965). Instead of forming part of a widespread species, distributed from East Africa to the Cape, we demonstrate that South African *Agabus* are all endemic to the region, and comprise a group of three semi-cryptic species, one of which is relatively widespread. These species constitute the southernmost records in the world for this otherwise largely Holarctic genus which is most diverse in the northern hemisphere and absent from South America and Australia (Miller and Bergsten 2016). The discovery of *Agabus anguluverpus* sp. nov. on Mount Kenya, shows that our knowledge of the *Agabus* fauna of high altitude areas in East Africa is also incomplete. Many of the mountain systems associated with the Rift remain poorly investigated for aquatic insects, and we suspect that additional, new, species of the genus remain undiscovered.

In his revision of the *raffrayi* species group, Nilsson (1992a) considered that the shape of male genitalia, although universally used for species-level identification in the genus elsewhere, was largely uninformative and consequently this character was not used in the determination key to the group. Here we show instead that the three distinct species found in South Africa differ from other *raffrayi* group species by having a preapically shorter male aedeagus. Coincidentally, this genitalic feature is also characteristic of the new species we describe from high elevations on Mount Kenya. We hypothesize, however, that a preapically prolonged aedeagus may be a synapomorphy of *A. ruwenzoricus* and its relatives, and that the shorter plesiomorphic state may not necessarily indicate a close relationship, at least between South African and Kenyan beetles.

## Supplementary Material

XML Treatment for Agabus raffrayi

XML Treatment for Agabus pallidus

XML Treatment for Agabus ruwenzoricus

XML Treatment for Agabus sjostedti

XML Treatment for Agabus dytiscoides

XML Treatment for Agabus anguluverpus

XML Treatment for Agabus austellus

XML Treatment for Agabus riberae

XML Treatment for Agabus agulhas
